# Compromise of Multiple Time-Resolved Transcriptomics Experiments Identifies Tightly Regulated Functions

**DOI:** 10.3389/fpls.2012.00249

**Published:** 2012-11-16

**Authors:** Sebastian Klie, Camila Caldana, Zoran Nikoloski

**Affiliations:** ^1^Genes and Small Molecules Group, Max Planck Institute of Molecular Plant PhysiologyPotsdam, Germany; ^2^Brazilian Bioethanol Science and Technology Laboratory (CNPEM/ABTLuS)Campinas, Brazil; ^3^Systems Biology and Mathematical Modeling Group, Max Planck Institute of Molecular Plant PhysiologyPotsdam-Golm, Germany

**Keywords:** transcriptomics time-series data, compromise of data tables, multi-way data analysis, Arabidopsis

## Abstract

With the advent of high-throughput technologies for data acquisition from different components (i.e., genes, proteins, and metabolites) of a given biological system, generation of hypotheses, and biological interpretations based on multivariate data sets become increasingly important. These technologies allow for simultaneous gathering of data from the same biological components under different perturbations, including genotypic variation and/or changes in conditions, resulting in so-called multiple data tables. Moreover, these data tables are obtained over a well-chosen time domain to capture the dynamics of the response of the biological system to the perturbation. The computational problem we address in this study is twofold: (1) derive a single data table, referred to as a *compromise*, which captures information common to the investigated set of multiple tables and (2) identify biological components which contribute most to the determined compromise. Here we argue that recent extensions to principle component analysis called STATIS and dual-STATIS can be used to determine the compromise on which classical techniques for data analysis, such as clustering and term over-enrichment, can be subsequently applied. In addition, we illustrate that STATIS and dual-STATIS facilitate interpretations of a publically available transcriptomics data set capturing the time-resolved response of *Arabidopsis thaliana* to changing light and/or temperature conditions. We demonstrate that STATIS and dual-STATIS can be used not only to identify the components of a biological system whose behavior is similarly affected due to the perturbation (e.g., in time or condition), but also to specify the extent to which each dimension of the data tables reflect the perturbation. These findings ultimately provide insights in the components and pathways which could be under tight control in plant systems.

## Introduction

High-throughput technologies are routinely applied to obtain a snapshot of plant systems operating under a given environmental condition. The resulting multivariate data sets gathered from the same set of biological entities (e.g., genes) under various conditions require the development of methods for simultaneous analysis of multiple data sets (or *data tables*). The investigated environmental conditions are usually not independent in the sense that some, but not necessarily all, of the controlled parameters are varied in the process of generating a pair of data tables. Therefore, there is an increasing need for development and application of multivariate statistical techniques which account for the inherent dependence between data tables while capturing what is common, i.e., preserved, across them.

The goal of this study is to introduce STATIS and dual-STATIS in the analysis and interpretation of transcriptomics data over the same set of genes under varying but not necessarily independent environmental conditions sampled at same time points starting from a well-defined reference. The idea of STATIS and dual-STATIS is based on integrating a given set of data tables into an optimum weighted average, called a *compromise*, which captures what is common to all or a subset of analyzed tables. The compromise is obtained based on principal component analysis (PCA) of a specially constructed matrix. Since we consider the case where no supervised information is available about the gene labels, it is then possible to apply classical unsupervised learning techniques to the resulting compromise. The approach presented in this study may be regarded as an instance of the *multi-way* unsupervised learning problem which requires decomposition of a multidimensional table (Geladi, 1989).

The classical way of investigating two-way data is by performing PCA on the two-dimensional matrix which is equivalent to singular value decomposition (SVD) of data matrix and the eigenvalue decomposition of the corresponding covariance matrix. Since their early application to analyses microarray gene expression data (Alter et al., 2000; Yeung and Ruzzo, 2001), PCA and SVD are routinely applied to reduce the dimensionality and posit biological hypotheses. The simplest extension of this method to multi-way data is application of PCA on the unfolded two-way data. The more advanced multi-way data analysis can be traced back to Hitchcock (1927), who investigated the three-mode PCA, generalized to N-mode PCA, which is commonly referred to as Tucker decomposition (Kroonenberg, 1983). A constrained version of Tucker decomposition is Canonical Polyadic Decomposition (CPD), known as PARAFAC (Harshman, 1970). Like PCA, PARAFAC, and Tucker decompose the multi-way data into sets of scores, known as loadings which potentially describe the data in a form closest to the original. In addition, Generalized Procrustes Analysis (GPA) and Simultaneous Components Analysis (SCA) can also be applied to integrate multiple data tables (Gower and Dijksterhuis, 2004). However, PARAFAC, GPA, and SCA are based on the alternating least squares algorithm which may result in a local optimum, and the problem formulation may have alternative optima (ten Berge, 1993; Kiers, 1998). Moreover, as more dimensions (i.e., ways) are included in the data set, it becomes more difficult to provide interpretation of the findings (Bro, 1997). Interestingly, STATIS and dual-STATIS construct the compromise space from the similarity structure of the considered tables and require application of generalized eigenvalue decomposition which overcomes the aforementioned drawbacks (Lavit, 1988). We would like to stress that STATIS does not operate on the actual tables but on the corresponding covariance matrices, and employs the extracted principal components to project the analyzed tables, variables, and observations.

Therefore, STATIS and dual-STATIS, when applied on transcriptomics data, directly relate to the problem of empirically estimating the covariance matrices in the case where the number of samples is small and the number of biochemical entities (i.e., genes) is large – typically arising in systems biology studies. In such a setting, the classical methods for covariance matrix estimation are highly unstable since the covariance matrix is likely singular. However, recent theoretical advances, some of which take advantage of the special types of data (e.g., time-series), facilitate more reliable estimates and allow their usage in conjunction with the methods applied in this study (Andrews and Monahan, 1992; Schaefer and Strimmer, 2005; Rudelson and Vershynin, 2010; Vershynin, 2012).

## Materials and Methods

### Data

**Figure 1:**
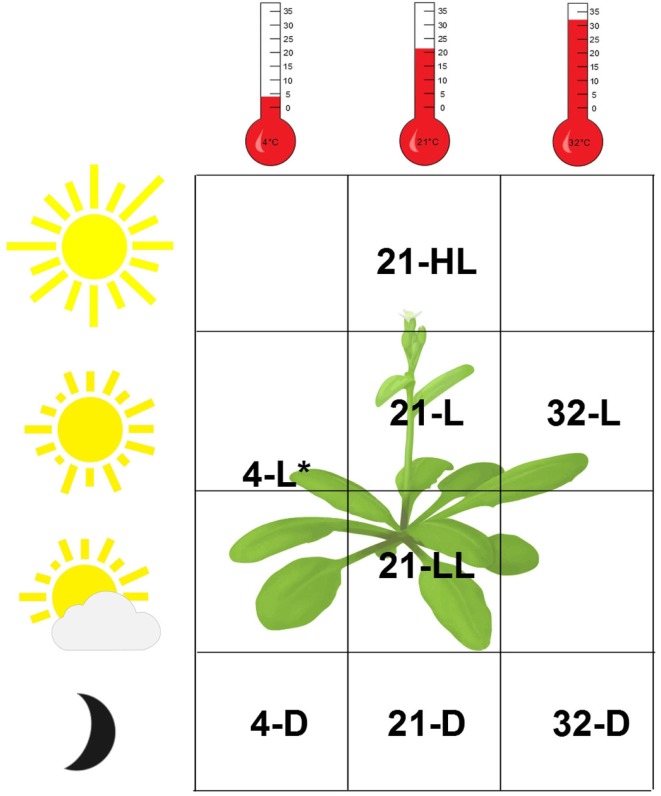
**Overview of the experimental conditions of the *Arabidopsis thaliana* data set**. Both light intensity (*D* = 0 μE, LL = 75 μE, *L* = 150 μE, and HL = 300 μE; *4-L = 80 μE) and temperature (4, 21, and 32°C) were varied, resulting in eight distinct environmental conditions including the control, denoted by 21-L.

We consider a recently obtained transcriptomics data set from *Arabidopsis thaliana* plants exposed to eight environmental conditions differing in light intensity and/or temperature (Caldana et al., 2011). The response to each condition was followed over 23 time points (including the zero time point) in 20 min intervals up to 360 min starting from the same reference. In addition, samples were taken after 5, 10, 640, and 1280 min. We note that the same set of time points, covering both a linear and a logarithmic scale, was used in all conditions. *Arabidopsis thaliana* plants, grown in soil at 21°C and 150 μEm^−2^s^−1^, were either kept at this condition or transferred to seven different environments reflecting a light gradient ranging from darkness to high-light stress and a temperature gradient from 4°C over 21 to 32°C. To identify similarities and differences between these data sets, either one (light or temperature) or both environmental parameters were changed. This resulted in the following seven (combinations of) environmental conditions: (*i*) 4°C and darkness (abbreviated as 4-D), (*ii*) 21°C and darkness (21-D), (*iii*) 32°C and darkness (32-D), (*iv*) 4°C and 85 μEm^−2^s^−1^ (light; 4-L), (*v*) 21°C and 75 μEm^−2^s^−1^ (low-light; 21-LL), (*vi*) 21°C and 300 μEm^−2^s^−1^ (high-light; 21-HL), and (*vii*) 32°C and 150 μEm^−2^s^−1^ (light; 32-L). Together with the set of plants kept at the original conditions (21°C and 150 μEm^−2^s^−1^, abbreviated as 21-L and referred to as control), eight different conditions were considered, as illustrated in Figure 1. Single RNA sample isolated from a pool of the six independent plants per condition and time point was used for transcript profiling with the Affymetrix ATH1 array, resulting in eight data tables including the time-resolved gene expression (Caldana et al., 2011).

As a starting point, we used the 15,089 genes analyzed in the original study after normalization and filtering of the microarray data using standard methods. The data set was subsequently filtered for genes which exhibit fold-change of at least two with respect to the first time point (0 min corresponding to the control condition) in at least two time points for any condition and a coefficient of variation within the considered time domain of at least one. These are reasonable criteria to remove less informative, and possibly non-differentially expressed, genes. This strategy was used as there is only one replicate for each time point, precluding application of more rigorous statistics. The preprocessing step resulted in identification of 2,276 genes which were used in the analysis based on STATIS and dual-STATIS.

### Overview of STATIS and dual-STATIS

Suppose that we are given *K* data tables *X*_1_, …, *X*_K_ corresponding to the investigated conditions, and let they be gathered as blocks of a matrix *X* = [*X*_1_|…|*X_K_*]. Moreover, let each one of these tables include observations on *n* genes, corresponding to the rows, over *t* time points, given by the columns. Furthermore, we will use $x_{ij}^{l}$ denote the expression of the *i*^th^ gene in the *j*^th^ time point of the *l*^th^ condition. In addition, each data table is preprocessed, i.e., centered and normalized according to the recommendation of Abdi et al. (2012) and Smilde et al. (2003). Each of the rows is assigned a non-negative mass *m*, so that the masses over all rows sum up to one. Here we used uniform values for the masses, resulting in a vector of masses ***m*** is of unit length.

In the first step of STATIS, the cross-product matrix $S_{i}=X_{i}X_{i}^{T}$ is calculated for every table *X_i_*, 1 ≤ *i* ≤ *K*. Each of the resulting cross-product matrices captures the relationship between the rows, also known as within-table structure, as seen by the corresponding table. To investigate the between-table structure, one then determines the matrix *C* in which the entry *c_ij_* equals the inner product of the cross-product matrices *S_i_* and *S_j_*, 1 ≤ *i,j* ≤ *K*. More concretely, $c_{ij}=\overset{t}{\underset{p=1}{\sum }}\overset{t}{\underset{q=1}{\sum }}S_{p,q}^{i}S_{p,q}^{j}$. The optimum weights for combining the data tables are then obtained from the first eigenvector of *C*, *u*_1_, which corresponds to the eigenvalue of largest weight. The entry $u_{i}^{1}$ of the first eigenvector captures to the similarity between the *i*^th^ table, 1 ≤ *i* ≤ *K*, and all other investigated data tables. Therefore, the quantity $\alpha_{i}=u_{i}^{1}∕∥u_{1}∥$ corresponds to the weights for the *i*^th^ table, so that $\overset{K}{\underset{i=1}{\sum }}\alpha_{i}=1$. The compromise cross-product matrix is then given by $S=\overset{K}{\underset{i=1}{\sum }}\alpha_{i}S_{i}$.

In the second step, generalized eigen value decomposition of the compromise *S* is performed, so that *S* = *P*Λ*P^T^* under the constraint *P^T^MP* = *I*, where *I* is the identity matrix. It is easy to show that the eigenvalue decomposition of *S* is equivalent to generalized SVD of *X*, since *S* = *PAP^T^* with *A* = diag(***a***), where ***a*** = [α_1_**1***^T^*|…|α*_K_***1***^T^*]. The loadings can then be computed as

$$Q=X^{T}MP\Delta^{-1},$$

where Λ = Δ^2^. The compromise factor scores, *F*, can be computed from *S* as

$$F=SMP\Delta^{-1}.$$

The loadings of the variables from table *i*, 1 ≤ *i* ≤ *K*, are obtained as

$$Q=X_{i}^{T}MP\Delta^{-1},$$

while the factor scores, *F*, for table *i*, 1 ≤ *i* ≤ *K*, are given by

$$F_{i}=S_{i}MP\Delta^{-1}.$$

In addition, similar to PCA, in STATIS one can determine the contribution of rows, columns, and tables to the principal components. The contribution of the *i*^th^ row to the component *b* is given by

$$ctr_{i,b}=\frac{m_{i}f_{i,b}^{2}}{\lambda_{b}},$$

where λ*_b_* is the *l*^th^ largest eigenvalue from the decomposition of the compromise *S* and *f_i,b_* denotes the factor score of the *i*^th^ observation for the *b*^th^ dimension. Since $\lambda_{b}=\underset{i}{\sum }m_{i}f_{i,b}^{2}$, all contributions take values between 0 and 1. The larger the contribution, the more the row contributes to the component. In order to base the interpretation of a given component on the rows that have significant contributions, a bootstrap procedure can be applied. Briefly, a bootstrap sample (i.e., a uniform random sample with replacement) is performed on the set of tables. By repeatedly conducting STATIS analyses on random samples of tables bootstrap contributions $ctr_{i,b}^{*}$ are obtained. Finally, these bootstrap estimates of contribution are transformed in bootstrap ratios, that can be interpreted in a similar way as a *t*-statistic (Abdi et al., 2012). Significantly contributing rows whose factor scores have different sign can then be contrasted to interpret the component. Similarly, based on the loading of the *j*^th^ variable for the *b*^th^ dimension, *q_j,b_*, the contribution of *j*^th^ column to the *b*^th^ component can be determined as

$$ctr_{j,b}=a_{j}q_{j,b}^{2}.$$

Likewise, the contribution of the *l*^th^ table can then be simply defined as the sum of the contributions of its variables:

$$ctr_{l,b}=\underset{j}{\sum }ctr_{j,b}.$$

The contribution of all tables of often referred to as the *interstructure* or inner product map (Lavit et al., 1994; Abdi et al., 2012). The contributions of columns and tables to a component have the same properties as the contributions of rows, described above; hence, they can be used in interpretation in a similar way. It is also important to identify tables that represent well the overall similarity with respect to all considered tables, i.e., to quantify their contribution to the compromise *S*. To this end, the investigation of table weights α*_i_* as well as the *R_V_* coefficient have been considered (Thioulouse et al., 1997). The *R_V_* coefficient is a measure of similarity of two cross-product matrices *S_k_* and $S_{k^{\prime }}$ both *n* × *n* matrices, defined as the inner product (Horn and Johnson, 2006).

$$R_{V}\left(S_{k},S_{k^{\prime }}\right)=\overset{t}{\underset{i}{\sum }}\overset{t}{\underset{j}{\sum }}S_{i,j,k}S_{i,j,k^{\prime }},$$

which takes values between 0 and 1 as both matrices are positive semidefinite and normalized, thus, being equal to the cosine of the two matrices (Abdi et al., 2012).

The analysis based on dual-STATIS can be carried our analogously on the transposed data table. In other words, in the first step of dual-STATIS, the cross-product matrix is calculated for every table *X_i_*, 1 ≤ *i* ≤ *K*, as $S_{i}=X_{i}^{T}X_{i}$. Each of the resulting cross-product matrices in the dual-STATIS setting thus captures the relationships between the columns of the original data tables, referred to as *within-table structure*. The subsequent steps of dual-STATIS are identical to the STATIS analysis, finally providing a compromise, *S*′, for the variables rather than the observations of the original data tables.

A reference implementation, on which our analysis is based on, is available in the ade4 package for the open source statistical software R (Gentleman and Rober, 1996; Thioulouse et al., 1997).

### Alternative data normalization strategy

Following considerations by Caldana et al. (2011) we consider a second normalization strategy for the whole time-course data set. Here, all transcript expression levels at every time point over all experimental conditions are normalized with respect to the corresponding control time point by subtracting the obtained fold-changes. This does not affect the control condition; however, it allows better assessment of the stress similarities and specificities by further eliminating circadian and diurnal responses as measured under control condition. We note that based on this normalization strategy, the previously outlined procedure for identifying informative genes now resulted in 3025 genes. We repeatedly apply both STATIS and dual-STATIS analysis to reveal and emphasize findings pertaining to diurnal changes.

## Results

### The contributions of conditions in the STATIS analysis reveal the influence of both temperature and light conditions on transcript levels

**Figure 2:**
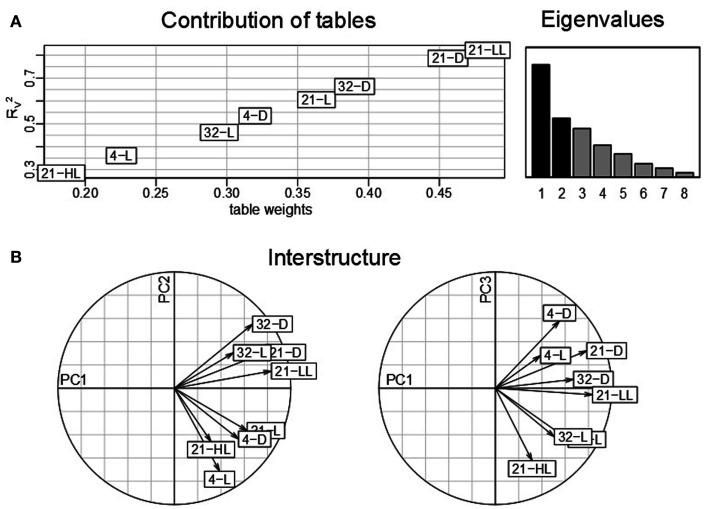
**STATIS analysis of the importance of tables, i.e., conditions**. **(A)** Table weights and *R_V_* coefficient allow assessing the influence of tables on the resulting compromise; the eigenvalues correspond to the variance captured by the principal components. **(B)** The interstructure is the projection of the individual condition on the principal components (PC1 and PC2 on the left, PC1 and PC3 on the right) allowing characterization of the effects governing the separation of conditions.

Similar to the classical PCA, analysis based on STATIS allows describing the contributions of tables, variables, and observations. Here, the *K* = 8 tables correspond to the eight environmental conditions obtained from the Arabidopsis experiment described in Section “Materials and Methods,” where the variables denote the 23 time points measured for each condition and the observations correspond to the 2,276 genes which are obtained using the aforementioned filtering strategy. An analysis of the contributions of the individual data tables to the compromise *S* then renders it possible to determine the condition which has the strongest influence on the determined principal components and further reflects good overall similarity to the other data tables from the remaining conditions. Figure 2A displays the obtained table weights α*_i_* for all conditions (*x*-axis), as well as the similarity quantified by the $R_{V}^{2}$ coefficient of the compromise and the individual tables (*y*-axis). One can observe that the low-light condition, 21-LL, as well as the 21-D, both at control temperature, have the strongest effect in the derivation of the compromise. This indicates that the transcriptomics changes under normal temperature regimes coupled with darkness/low-light conditions are characteristic for the entire data set, thus, superimposing the changes in the remaining combinations of conditions. Moreover, the light stress (21-HL) and cold stress condition (4-L) have the lowest influence within the complete dataset reflected by the low $R_{V}^{2}$ coefficient and table weight.

Subsequent investigations of the table interstructure, i.e., the contribution of the tables to the first, second, and third principal components (Figure 2B, left and right) further corroborates this finding: With exception of the aforementioned light stress condition (21-HL), the remaining light regimes at control temperature conditions, namely, 21-LL, 21-D, and 21-L, exhibit strong contributions to the first principal component. Changes in ambient temperature, e.g., cold and heat, contribute reciprocally to the second principal component. Additionally, the second principal component separates the light conditions at control temperatures (21-D and 21-L). Finally, the effect of the diurnal rhythm is captured in the third principal component. Here, the darkness conditions, namely, 4-D, 21-D, and 32-D, contribute positively, while the high-light condition, 21-HL, does so negatively. Interestingly, the data table corresponding to the combination of cold stress and light, i.e., 4-L, exhibits a similar contribution to the third principal component as the other darkness conditions. This observation could be partially attributed to the fact that the light intensity at 4°C is lower (85 μE) than the others “L” controls (150 μE) as an attempt to avoid light stress. Furthermore, this might be related to the attenuating effect of the low temperature as previously reported by Caldana et al. (2011) as well as a general stress response, since both extended dark and cold conditions have been widely reported to affect plant growth.

Furthermore, the first principal component in the interstructure characterizes the dominant transcriptional response within the complete dataset as it corresponds to the largest eigenvalue which is proportional to the variance explained. The strong effect on the covariance structure of transcripts in all datasets under the control temperature condition can be attributed to the experimental design; namely, out of eight environmental conditions, four were conducted at different light regimes and the same temperature of 21°C. Moreover, the second strongest effect, corresponding to the contributions to the second principal component is the contrast of cold and heat stress, while the third strongest influence is ascribed to the diurnal cycle.

**Figure 3:**
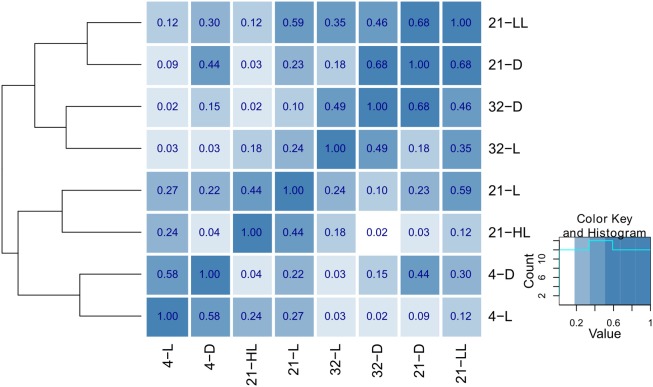
**Heatmap and hierarchical clustering illustrating the similarity of the eight environmental conditions**. The pairwise similarity is derived using the *R_V_* coefficient of the corresponding cross-product matrices obtained for each condition. The hierarchical clustering was obtained using average linkage on the pairwise distances obtained using 1 − *R_V_*.

Apart from the contributions of individual tables to the compromise, the pairwise cosine similarity, i.e., the *R_V_* coefficient, can be used to further assess the similarities between the tables. Figure 3 illustrates the similarity between conditions as a heatmap (based on the function heatmap.2() in the gqplot R package). It becomes apparent that the type of conditions, in all considered combinations of perturbations, has an effect on the clustering: Primarily, similar temperature conditions (4, 21, and 32°C) cluster together (as derived by the cluster dendrogram) in comparison to conditions in the same light regime (4-D and 4-L and 32-D and 32-L, respectively). Under control, the secondary effect of light intensity can be seen by the co-clustering of the low-light/dark (21-LL, 21-D) and high-light/normal light (21-HL, 21-L) conditions.

In summary, these results demonstrate the advantages of the multi-table analysis based of STATIS as compared to classical PCA. In the classical PCA, distinct dimensions, corresponding to experimental factors, of the investigated data tables cannot be separately investigated. The reason is that individual table structure gets lost, since a combination of environmental conditions and time points, e.g., 21-L 60 min, serves as variables (often referred to as *loadings*) in a PCA. The analysis by STATIS however, allows the characterization of each of the eight experimental conditions independently of the time-factor. A similar consideration of the same dataset by classical PCA – as conducted in Figure 2A in Caldana et al. (2011) – displays each condition by all of its measured time points resulting in 184 variables in the loadings plot.

### Analysis of time points suggests presence of circadian/diurnal effects in light conditions and starvation response in dark conditions

**Figure 4:**
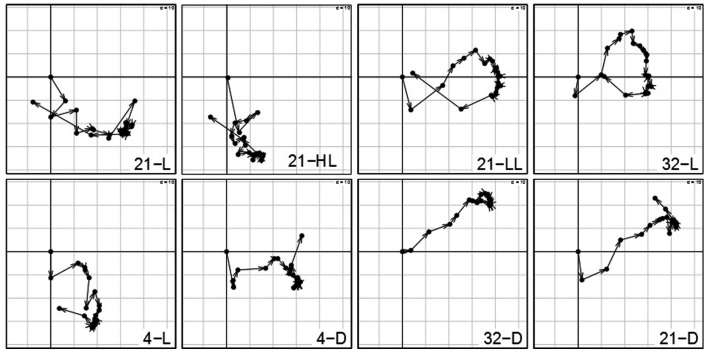
**Contributions of variables (i.e., time points 0–1280 min) by projection of the corresponding columns on the first and second principal components for each condition (panel 1–8) obtained by using the analysis based on STATIS**. As the first time point (0 min) is the same across conditions, it resides at the origin. Arrows/trajectories illustrate to temporal progression of contributions of the variables.

The analysis of the contributions of variables reflects the influence of the particular time point with respect to the overall transcriptional adjustments of the Arabidopsis in response to the applied stresses. Figure 4 shows the contributions of each time point per condition on the first (*x*-axis) and second (*y*-axis) principal component. Arrows between two successive time points illustrate the sequential progression of the contributions over time with the compromise space defined by the first two principal components. While all the trajectories of time points >0 min show substantial contribution to either the first or second principal component – clearly illustrating the ongoing transcriptional adjustments of *Arabidopsis*’ transcriptome – three observations are of particular interest:

(1) Even under the control condition, 21-L, we observed strong changes and varying influence on the compromise of the similarity structure in expression levels. One example is the expression of the central oscillator gene TOC1 (At5g61380; Strayer et al., 2000) that showed over fivefold – increase in the course of the control condition (Caldana et al., 2011) which reflects diurnal regulations as the time-series consider 22 h (the last time point is 1280 min).
(2) Within all light conditions, 21-L, 21-HL, 21-LL, and 32-L, except for cold, 4-L, the contribution of the late time points (especially 1280 min) is similar to the early time points indicating a conserved covariance structure of *Arabidopsis*’ transcript profiles in the beginning and final measurements. As the last time point almost comprises a complete diurnal cycle, this could be explained by circadian rhythm which is self-sustained despite the continuous light (McClung, 2006; Espinoza et al., 2010). Moreover, this effect persists in an alleviated form even at low and high temperatures (4 and 32°C) accounting for the temperature compensation of the circadian rhythm (McClung, 2008).
(3) In contrast, all three darkness conditions, 21-D, 4-D, and 32-D, do not exhibit this behavior; the later time points do not converge toward the early time points explained by a strong starvation effect due to the absence of light. Furthermore, the temperature (dominance on the second principal component; here, *y*-axis) has an effect on the early-mid time points of all three darkness conditions: the higher the temperature, the faster early-mid time points exhibit positive contribution on the second principal component. Particularly the 1280 min time point in cold, 4-D, is the only time point exhibiting a positive contribution, which again can be attributed to the previously mentioned attenuation effect of low temperature, whereas the exclusive positive contribution of time points in heat stress and darkness, 32-D, display the effects of a synergistic stress response. Finally, control temperature at darkness, 21-D, displays an intermediate profile. These observations corroborate the previous findings that darkness clearly leads to dramatic differences in the responses to temperature but these are not as striking as the effect of temperature on the response to darkness (Vershynin, 2012).

### Investigation of genes by STATIS reveals key pathways affected by the applied conditions

**Figure 5:**
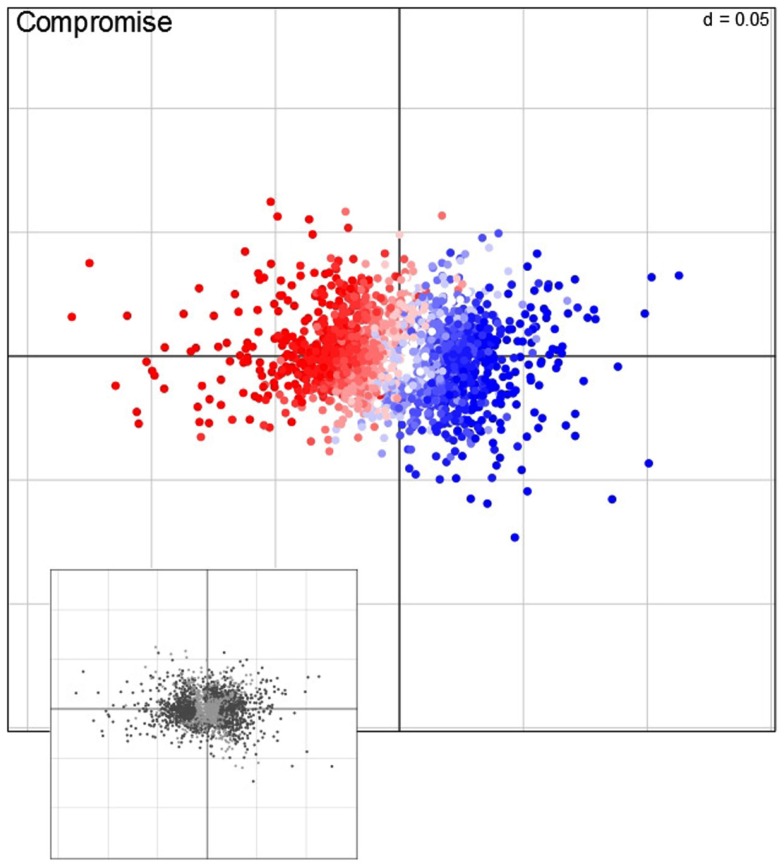
**Visualization of the compromise**. Each point represents one of the 2,276 genes, i.e., observations, projected on the first and second principal component. Red (repression) and blue (induction) color corresponds to the direction average fold-change of expression levels (main panel); the color intensity correspond to the magnitude of change. Genes that exhibit a significant contribution, as determined by bootstrapping procedures, are colored in dark, non-significantly contribution genes in light gray (lower panel).

Characterization of observations is a pivotal step of any descriptive statistical method. We note that in our setting, the observation correspond to the 2,276 genes. To this end, STATIS allows not only the investigation of the contribution of tables (i.e., conditions) and variables (i.e., time points), but also the analysis of the contributions of observations to the principal components. In particular, for the purpose of visualization, contributions of individual observations, rows in matrix *X*, are obtained by projection onto the principal components of the compromise. Figure 5 displays the contribution of genes to the first two principal components. Based on the projection of observation on the compromise, one can notice that the sign of the contribution of genes to the first principal component accounts either for the repression (red points) or the induction (blue) of the respective transcripts.

In order to determine which key pathways are associated with the genome-wide adjustments of gene expression, a gene set enrichment analysis (GSEA; Subramanian et al., 2005) of ontology terms is typically performed. However, an important intermediate step is to identify the observations (i.e., genes) which contribute significantly to either principal component. Here, bootstrap ratios (*cf*. Materials and Methods) are used to identify significant contributions at a significance level of 1%. Those genes (1371 in total) which are found to be significantly contributing to either the first or second principal component (illustrated as dark points in the small panel in Figure 5) are subjected to a GSEA by using MapMan terms (Thimm et al., 2004). Table 1 gives an overview of the MapMan terms (Version 1.1, January 2010^1^) found to be over-represented at a significance level of 1% [enrichment determined by hypergeometric distribution (Rivals et al., 2007) and a FDR corrected *p*-value ≤0.01 cutoff (Benjamini and Hochberg, 1995)]. By carefully inspecting Table 1, it is becomes apparent that when plants sense changes in the environment (i.e., light and temperature), several signal transduction pathways are triggered to adjust to the “new” condition as illustrated by the over-enrichment of MapMan bins such as “*hormone metabolism”* – brassinosteroid, ethylene, and ABA, “*receptor kinases*,” “*DNA synthesis/chromatine structure. histone*,” “*RNA regulation of transcription*” and “*micro RNA*.”

**Table 1:** **Overview of over-represented MapMan bins determined by an enrichment analysis using significantly contributing observations (i.e., genes) in the STATIS analysis at a significance level of 1%**.

MapMan bin	Description
1.1.1	PS.lightreaction.photosystem II
1.1.30	PS.lightreaction.state transition
1.2.4.1	PS.photorespiration.glycinecleavage.P subunit
2.1.2	Major CHO metabolism.synthesis.starch
2.2.2.1	Major CHO metabolism.degradation.starch.starch cleavage
3.2.3	Minor CHO metabolism.trehalose.potential TPS/TPP
3.4.3	Minor CHO metabolism.myo-inositol.InsP Synthases
10.6.2	Cell wall.degradation.mannan-xylose-arabinose-fucose
10.7	Cell wall.modification
13.1.4.1.4	Amino acid metabolism.synthesis.branched-chain group.common.branched-chain amino acid aminotransferase
13.1.6.3.1	Amino acid metabolism.synthesis.aromaticaa.phenylalanine.arogenate dehydratase/prephenatedehydratase
14.15	S-assimilation.AKN
14.2	S-assimilation.APR
16.2.1	Secondary metabolism.phenylpropanoids.lignin biosynthesis
16.5.1.1.1	Secondary metabolism.sulfur-containing.glucosinolates.synthesis.aliphatic
16.5.1.1.4	Secondary metabolism.sulfur-containing.glucosinolates.synthesis.shared
16.5.1.2.1	Secondary metabolism.sulfur-containing.glucosinolates.regulation.aliphatic
16.8.1	Secondary metabolism.flavonoids.anthocyanins
16.8.2	Secondary metabolism.flavonoids.chalcones
16.8.3	Secondary metabolism.flavonoids.dihydroflavonols
17.1.1.1.10	Hormone metabolism.abscisic acid.synthesis-degradation.synthesis.9-cis-epoxycarotenoid dioxygenase
17.3.1.2.2	Hormone metabolism.brassinosteroid.synthesis-degradation.sterols.SMT2
17.5.2	Hormone metabolism.ethylene.signal transduction
18.4.1	Co-factor and vitaminemetabolism.pantothenate.branched-chain amino acid aminotransferase
26.21	Misc.protease inhibitor/seed storage/lipid transfer protein (LTP) family protein
26.25	Misc.sulfotransferase
26.3.2	Misc.gluco-, galacto- and mannosidases.beta-galactosidase
26.4.1	Misc.beta 1,3 glucanhydrolases.glucan endo-1,3-beta-glucosidase
26.8	Misc.nitrilases, *nitrile lyases, berberine bridge enzymes, reticuline oxidases, troponinereductases
26.9	Misc.glutathione S transferases
27.3.50	RNA.regulation of transcription.General Transcription
27.3.6	RNA.regulation of transcription.bHLH,Basic Helix-Loop-Helix family
27.3.80	RNA.regulation of transcription.zf-HD
28.1.3	DNA.synthesis/chromatin structure.histone
29.5.4	Protein.degradation.aspartate protease
30.2.17	Signaling.receptorkinases.DUF 26
32	Micro RNA, natural antisense etc
34.13	Transport.peptides and oligopeptides

*MapMan bins are sorted based on their bin number*.

Following this response, as a result of both diurnal changes and light/dark contrasts, the carbon/nitrogen (C/N) metabolism is clearly affected. This response is reflected by the over-enrichment of MapMan bins associated to photosynthesis-related processes such and *“PS light reactions*” (darkness; bins 1.1.1; 1.1.30) followed by an over-enrichment of bins involved in sugar metabolism. For instance, starch synthesis (high and normal light) and degradation (darkness) as a result of nutrient limitation, e.g., bin 2.1.2 “*major CHO metabolism.synthesis.starch*” and 2.2.2 “*major CHO metabolism. degradation.starch*.” The metabolism of minor sugars, such as members of the raffinose (cold) family and trehalose (darkness) which are considered to act as signal under stress or energy deprivation, are also affected (bins 3.1 and 3.2). High-light (21-HL) results in over-enrichment of bins involved in “*PS.photorespiration*” and detoxification such as “*misc. gluthatione*.”

The C/N imbalance at darkness conditions is further supported by the over-enrichment of bins involved in nitrogen metabolism and nutrient recycling such as the bins containing genes related to “*Amino acid metabolism*” and “*Protein degradation*.” Next to the C/N balance disruption, under limited nutritional condition, like darkness, remobilization of carbon and nitrogen can occur through the secondary metabolism, as it is reflected by the over-enrichment of the bins involved in “*secondary metabolism.phenylpropanoid*,” “*secondary metabolism.glucosinolates*,” “*secondary metabolism.flavonoids*.” Additionally, bins 14.15 “*S-assimilation.AKN”* and “*misc.nitrilases*” are over-enriched as a result of early changes of abundance of the metabolite O-acetylserine (OAS), which is in accordance with existing results analyzing changes of OAS level within this data set as well as providing further evidence that OAS is a signaling molecule [aside from its role in sulfur assimilation (Nikiforova et al., 2005) based on further studies of transgenic plants (Hubberten et al., 2012)]. Altogether, changes in C/N balance affect plant growth. Plant growth is also closely regulated by cell wall expansion which requires constant remodeling of its components. We observed several MapMan bins involved in the cell wall remodeling might play a role in the remobilization of sugar in response to sugar starvation (Lee et al., 2007). Transcriptional changes in genes related to cell wall modification, synthesis, and degradation under starvation or dark treatment has been associated with the putative role of cell wall as alternative energy source and as a means of restricting cell growth and elongation (Baena-Gonzalez and Sheen, 2008).

Finally, it is important to realize that these findings are based on genes of significant contribution to the first and second principal component of the compromise of eight environmental conditions and thus represent the common aspects of *Arabidopsis*’ transcriptomic response under light and temperature changes.

### Dual-STATIS analysis demonstrates a conserved gradual progression of Arabidopsis’ transcriptomic adjustments in the time domain

**Figure 6:**
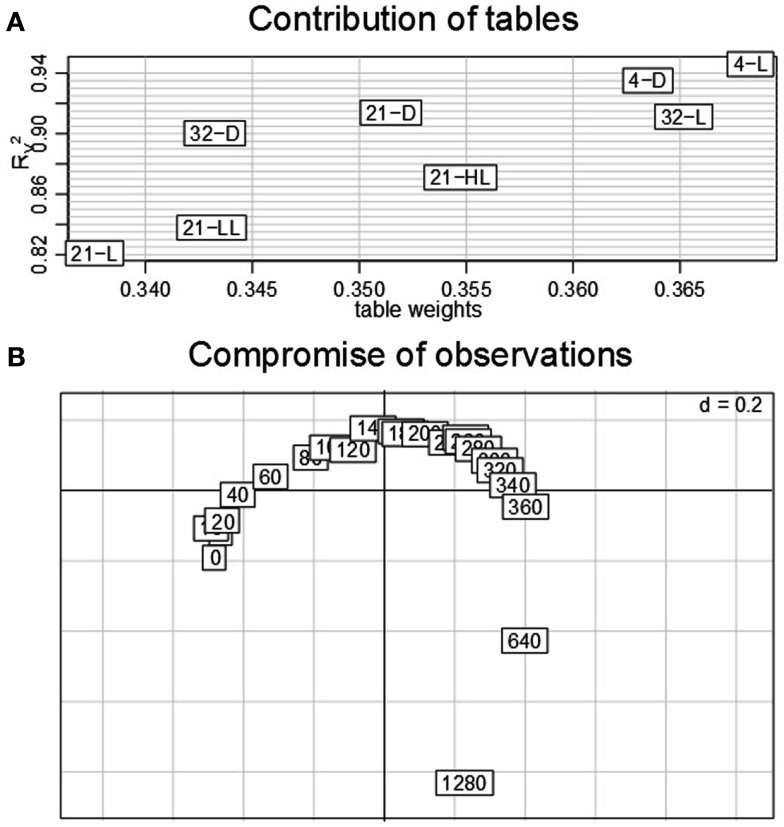
**Dual-STATIS analysis of the data set**. Here, the tables are transposed in order to characterize each condition by the time points (in this setting corresponding to observations). **(A)** Table weights and *R_V_* coefficient discriminate the influence of tables. **(B)** The contribution of the individual time points (0–1280 min) to the principal components allows describing to progression of transcriptional adjustments in Arabidopsis over the complete time-course.

The previously performed three steps of a STATIS analysis can be complemented by a dual-STATIS analysis. As outlined in Section “Materials and Methods,” the analysis based on dual-STATIS employs the transposed transcriptomic data set (i.e., in each table, a row now corresponds to a time point, while a column stands for a gene). Therefore, it enables to obtain a compromise based on the covariance between time points. Figures 6A,B display the obtained table weights and the *R_V_* coefficients, as well as the compromise of time points. We observe that – aside from minor differences – all eight experimental conditions exhibit comparable table weights (within the range of ∼0.34 to ∼0.37) and *R_V_* coefficients (within the range of 0.83–0.94). This indicates that the covariance of time points is similar under all experimental conditions suggesting similar temporal progression of transcriptional regulation.

Moreover, the contribution of time points, by projecting the corresponding rows onto the principal components of the compromise, displays an almost perfect cycle. With the exception of some intermediate time points (∼120–280 min) as well as the last time point (1280 min), all observations contribute equally to either the first or second principal component as indicated by their equidistant displacement from the origin. Such representation could be explained by a gradually progressing system under tight control of diurnal rhythm. Interestingly, these results contrast the anticipation of stronger contributions of early time points as a result of fast system-wide adaptations to environmental changes.

### STATIS analysis reveals that alternative expression data normalization strategies emphasize temperature and light stress responses

**Figure 7:**
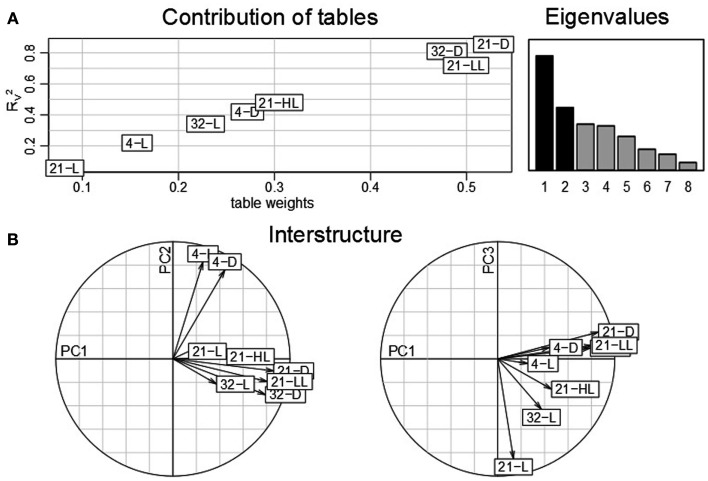
**STATIS analysis of the importance of tables, i.e., conditions of the re-normalized dataset**. **(A)** Table weights and *R_V_* coefficient allow assessing the influence of tables on the resulting compromise; the eigenvalues correspond to the variance captured by the principal components **(B)** The interstructure is the projection of the individual condition on the principal components (PC1 and PC2 on the left, PC1 and PC3 on the right) allowing characterization of the effects governing the separation of conditions.

The obtained findings pointed out that the control condition, 21-L, exhibits a considerable effect with respect to the contribution of conditions as well as time points (*cf*. Figures 2 and 4). Caldana et al. (2011) suggested to normalize the whole time-course for each stress to the respective time points measure under control condition to better assess the stress similarities and specificities further eliminating circadian and diurnal responses. Therefore, we repeated the analysis based on STATIS with a correspondingly re-normalized data set (*cf*. Material and Methods section): Figure 7A displays table weights and the coefficient of the compromise and the individual tables. In contrast to Figure 2A), now, the control condition has the least influence and, thus, the smallest similarity to the compromise. Furthermore, heat and control temperature conditions under low-light/darkness exhibit higher similarities to the compromise. A subsequent investigation of the interstructure (Figure 7B) shows that low-light/darkness conditions contribute the most to the first principal component, which captures differences among the light regimes across the eight experimental experiments. Moreover, the second principal component corresponds to temperature differences, since the low temperature conditions, 4-L and 4-D, contribute positively, while the high temperature experiments, 32-L and 32-D, contribute negatively. Finally, the third principal component facilitates the separation between the control, 21-L, and the remaining conditions.

**Figure 8:**
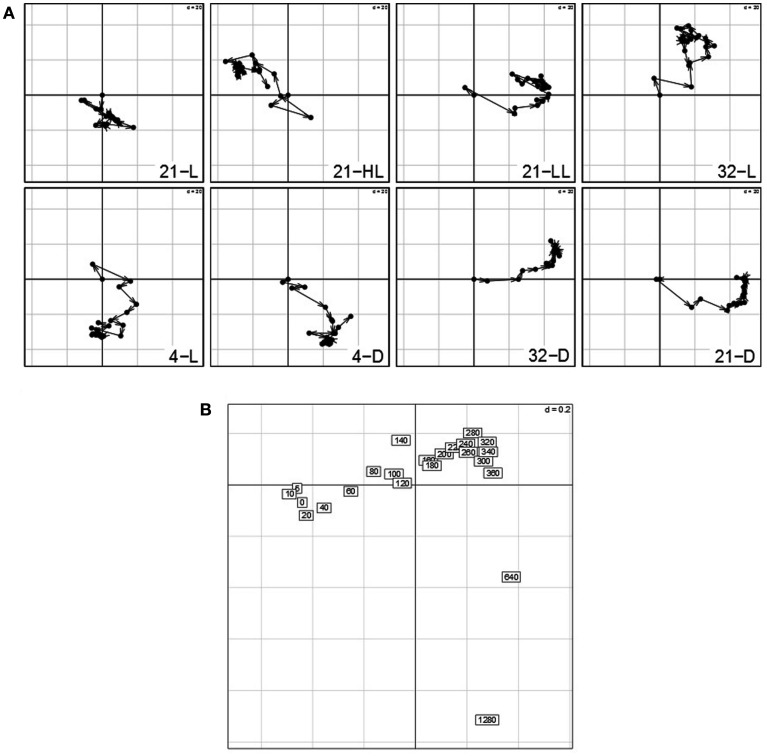
**(A)** Contributions of variables (i.e., time points 0–1280 min) by projection of the corresponding columns on the first and second principal components for each condition (panel 1–8) obtained by using the re-normalized dataset. As the first time point (0 min) is the same across conditions, it resides at the origin. Arrows/trajectories illustrate to temporal progression of contributions of the variables. **(B)** Dual-STATIS analysis of the transposed and re-normalized dataset.

The analysis of the contributions of variables, i.e., time points, additionally emphasizes the diminished effect of circadian regulation within the control condition (Figure 8A): as compared to all other seven conditions, the trajectories of the time point in 21-L display the weakest variations and contributions to either principal component. This observation is further confirmed by the fact that except from the high-light and the control conditions, 21-HL and 21-L, all others do not exhibit the aforementioned closing of the cycle with the last time point (1280 min). Similar to the interstructure, the contribution of the light regime to the first principal component (low-light/darkness positive; high-light negative) becomes more pronounced. Likewise, time points from the experiments in low temperature exhibit negative contributions, whereas those from high temperature experiments show positive contributions to the second principal component.

By performing an analysis based on dual-STATIS on the transposed and normalized data set, in which observations correspond to time points, *Arabidopsis*’ stress response becomes apparent (Figure 8B). In contrast to the temporal progression observed in the previously used dataset (*cf*. 4b), early time points, e.g., 5, 10, 20 40, 60 min, are grouped together in a non-sequential manner further separated from successive time points indicating the systems response to perturbation. This is additionally illustrated by considering the length of the trajectories between early time points in Figure 8A, which are generally of greater length than trajectories between two later time points.

From these observations, it becomes apparent that with limited presence of diurnal effects, the influence of observations, i.e., genes, allows a more detailed picture of the pathways affected by stress adjustments than the precious analysis. Table 2 displays the MapMan bins that are additionally enriched by performing a GSEA on highly contributing observations by using the normalized dataset (note that all MapMan bins exhibiting over-enrichment are also found using the normalized dataset). For instance, MapMan (sub) bins 2.1.2.2, 2.1.2.3, and 2.2.1.4 corresponding to “*major CHO metabolism.synthesis.starch.starch synthase*,” “*major CHO metabolism. synthesis.starch.starch branching*,” and “*major CHO metabolism.degradation. sucrose.hexokinase*” characterize in greater detail. Here, the effects of darkness and high-light conditions reciprocally lead to degradation of starch stored transiently in the chloroplasts or increase of synthesis as primary product of photosynthesis in leaves (Zeeman et al., 2007). As a second example, two more effects on transcriptional regulation become visible – MapMan bin 27.3.3 “*RNA.regulation of transcription.AP2/EREBP, APETALA2/Ethylene-responsive element binding protein family*” and 27.3.32 “*RNA.regulation of transcription.WRKY domain transcription factor family*.” The WRKY family of transcriptions factors, with up to 100 representatives in Arabidopsis (Eulgem et al., 2000), has been shown to be involved in response to abiotic stresses in plants (Chen et al., 2012). More specifically, one member, WRKY22 (AT4G01250), is exclusively induced over four, three, and fivefold in the three darkness conditions, 21-D, 4-D, and 32-D, respectively, and has been shown to regulate darkness-induced leaf senescence in *Arabidopsis* (Zhou et al., 2011). As a summary, the analysis of contributions of tables, variables, and observations by application of STATIS provides insights for different dimension of the data tables (i.e., conditions, time points, and genes). Moreover, the STATIS analysis on the re-normalized data allows exposing those biological components which remain conserved across all conditions, by subsequently reducing typically preserved dynamics of the organism, e.g., diurnal behavior, by application of different normalization strategies.

**Table 2:** **Overview of over-represented MapMan bins using significantly contributing observations (i.e., genes) in the STATIS analysis of the re-normalized dataset**.

MapMan bin	Description
1.1.5.4	PS.lightreaction.other electron carrier (ox/red).ferredoxinoxireductase
2.1.2.2	Major CHO metabolism.synthesis.starch.starch synthase
2.1.2.3	Major CHO metabolism.synthesis.starch.starch branching
2.2.1.4	Major CHO metabolism.degradation.sucrose.hexokinase
2.2.2.3	Major CHO metabolism.degradation.starch.glucan water dikinase
8.1.9	TCA/org transformation.TCA.malate DH
10.2	Cell wall.cellulose synthesis
10.6.3	Cell wall.degradation.pectatelyases and polygalacturonases
10.8.99	Cell wall.pectin*esterases.misc
11.6	Lipid metabolism.lipid transfer proteins etc
16.2.1.3	Secondary metabolism.phenylpropanoids.lignin biosynthesis.4CL
21.2.1.2	Redox.ascorbate and glutathione.ascorbate.GDP-l-galactose-hexose-1-phosphate guanyltransferase
23.5.4	Nucleotide metabolism.deoxynucleotidemetabolism. ribonucleoside-diphosphatereductase
26.10	Misc.cytochrome P450
26.2	Misc.UDPglucosyl and glucoronyltransferases
26.6	Misc.O-methyl transferases
27.3.3	RNA.regulation of transcription.AP2/EREBP, APETALA2/Ethylene-responsive element binding protein family
27.3.32	RNA.regulation of transcription.WRKY domain transcription factor family
29.2.1.1.3.2.35	Protein.synthesis.ribosomalprotein.prokaryotic. unknown organellar.50S subunit.L35
33.1	Development.storage proteins
34.2	Transporter.sugars

*Note, that this table contains bins which are derived in addition to those displayed in Table 1. A significance level of 1% was used for the enrichment analysis and MapMan bins are sorted based on their bin number*.

## Discussion

To capture the complex behavior of organisms, systems biology studies require design of experiments and analysis of the corresponding read-outs, whereby multiple components and subsystems are simultaneously affected upon various internal (i.e., structural) and/or external (i.e., condition) perturbations. Descriptive and inferential statistical analysis of such multidimensional data sets, usually including observation of biochemical entities (e.g., genes) whose number is comparatively larger than that of observations, necessitate the development and application of novel methods. The principle requirement for such methods is that they provide the possibility for simultaneous analysis of all or some of the multidimensional data sets, while inferring the biochemical entities which most contribute to the resulting observations.

The methods used in this study, namely, STATIS and dual-STATIS, derive a single table, referred to as a *compromise*, which captures information common to the investigated multiple data sets. We note that the compromise is derived as a linear combination of the covariance matrices corresponding to the individual data sets, with coefficient obtained from the eigenvalue decomposition of a special matrix capturing the congruence for all pairs of data sets. Moreover, since (dual-)STATIS can be seen as a form of generalized SVD, of which PCA is a very special instance, the contribution of each row and/or column of each data set to the derived compromise can be investigated through several projections. Moreover, classical statistical techniques, such as bootstrapping and jackknifing, can be used to infer which of the contributions are statistically significant.

Here we illustrated the usage of STATIS in the analysis of time-resolved transcriptomics data sets obtained from *Arabidopsis thaliana* under combination of growth conditions. Analysis based on the coefficient of congruence, i.e., the *R_V_* coefficient, for a pair of tables is in agreement with the established biological knowledge regarding the influence of mild vs. strong perturbation on the level of transcripts, with temperature perturbation having a larger effect on the similarity of the data sets in comparison to the modulation of light. In addition, our detailed examination of time points indicates the circadian/diurnal effects in light conditions, while starvation response is pronounced in dark conditions. Finally, we demonstrated that dual-STATIS can be integrated with GSEA to determine gene functions most affected by the considered conditions by using projections onto the compromise, itself capturing the similarities across the data sets. As with PCA, the choice of which components are to be used plays an important role in the interpretation of the data.

Our novel analysis of transcriptomics data sets based STATIS and dual-STATIS raises some issues which could likely be addressed through modification of the applied methods. For instance, the usage of covariance matrix does not allow the consideration of the time domain implicitly present in time-resolved data nor it is suitable for categorical variables. To this end, extension to STATIS, called DISTATIS, may facilitate the treatment of other distance measures in creating the compromise, which will be explored in a future study. Finally, one may consider extension of the illustrated methods so as to allow generation of a compromise data table. Clearly, moving away from a compromise on the level of the similarity structure within each data set may bring the modified approach closer to multi-way data analysis.

## Conflict of Interest Statement

The authors declare that the research was conducted in the absence of any commercial or financial relationships that could be construed as a potential conflict of interest.

## Footnotes

^1^http://mapman.gabipd.org/

## References

[B1] Abdi H. Williams L. J. Valentin D. Bennani-Dosse M. (2012). STATIS and DISTATIS: optimum multi-table principal component analysis and three way metric multidimensional scaling. Wiley Interdiscip. Rev. Comput. Stat. 4, 124–167 10.1002/wics.198

[B2] Alter O. Brown P. O. Botstein D. (2000). Singular value decomposition for genome-wide expression data processing and modeling. Proc. Natl. Acad. Sci. U.S.A. 97, 10101–10106 10.1073/pnas.97.18.10101 10963673 PMC27718

[B3] Andrews D. W. K. Monahan J. C. (1992). An improved heteroskedasticity and autocorrelation consistent covariance matrix estimator. Econometrica 60, 953–966 10.2307/2951574

[B4] Baena-Gonzalez E. Sheen J. (2008). Convergent energy and stress signaling. Trends Plant Sci. 13, 474–482 10.1016/j.tplants.2008.06.006 18701338 PMC3075853

[B5] Benjamini Y. Hochberg Y. (1995). Controlling the false discovery rate: a practical and powerful approach to multiple testing. J. R. Stat. Soc. Series B Stat. Methodol. 57, 289–300

[B6] Bro R. (1997). PARFAC. Tutorial and Applications, Vol. 38 Amsterdam: Elsevier

[B7] Caldana C. Degenkolbe T. Cuadros-Inostroza A. Klie S. Sulpice R. Leisse A. (2011). High-density kinetic analysis of the metabolomic and transcriptomic response of Arabidopsis to eight environmental conditions. Plant J. 67, 869–884 10.1111/j.1365-313X.2011.04640.x 21575090

[B8] Chen L. Song Y. Li S. Zhang L. Zou C. Yu D. (2012). The role of WRKY transcription factors in plant abiotic stresses. Biochim. Biophys. Acta 1819, 120–128 10.1016/j.bbagrm.2011.09.002 21964328

[B9] Espinoza C. Degenkolbe T. Caldana C. Zuther E. Leisse A. Willmitzer L. (2010). Interaction with diurnal and circadian regulation results in dynamic metabolic and transcriptional changes during cold acclimation in Arabidopsis. PLoS ONE 5, e14101 10.1371/journal.pone.0014101 21124901 PMC2990718

[B10] Eulgem T. Rushton P. J. Robatzek S. Somssich I. E. (2000). The WRKY superfamily of plant transcription factors. Trends Plant Sci. 5, 199–206 10.1016/S1360-1385(00)01600-9 10785665

[B11] Geladi P. (1989). Analysis of multi-way (multi-mode) data. Chemometr. Intell. Lab. Syst. 7, 11–30 10.1016/0169-7439(89)80108-X

[B12] Gentleman R. I. Rober R. (1996). A language for data analysis and graphics. J. Comput. Graph. Stat. 5, 299–314 10.2307/1390807

[B13] Gower J. C. Dijksterhuis G. B. (2004). Procrustes Problems. New York: Oxford University Press, 233

[B14] Harshman R. A. (1970). Foundations of the PARAFAC procedure: model and conditions for an “explanatory” multi-mode factor analysis. UCLA Working Pap. Phon. 16, 1–84

[B15] Hitchcock F. L. (1927). The expression of a tensor or a polyadic as a sum of products. J. Math. Phys. 6, 164–189

[B16] Horn R. A. Johnson C. R. (2006). Matrix Analysis. Cambridge: Cambridge University Press

[B17] Hubberten H. M. Klie S. Caldana C. Degenkolbe T. Willmitzer L. Hoefgen R. (2012). Additional role of O-acetylserine as a sulfur status-independent regulator during plant growth. Plant J. 70, 666–677 10.1111/j.1365-313X.2012.04905.x 22243437

[B18] Kiers H. A. L. (1998). A three–step algorithm for CANDECOMP/PARAFAC analysis of large data sets with multicollinearity. J. Chemometr. 12, 155–171 10.1002/(SICI)1099-128X(199805/06)12:3<155::AID-CEM502>3.0.CO;2-5

[B19] Kroonenberg P. M. (1983). Three-Mode Principal Component Analysis. Theory and Applications. Leiden: DSWO Press

[B20] Lavit C. (1988). Analyse Conjointe de Tableaux Quantitatifs. Paris: Masson

[B21] Lavit C. Escoufier Y. Sabatier R. Traissac P. (1994). The ACT (STATIS method). Comput. Stat. Data Anal. 18, 97–119 10.1016/0167-9473(94)90134-1

[B22] Lee E. J. Matsumura Y. Soga K. Hoson T. Koizumi N. (2007). Glycosyl hydrolases of cell wall are induced by sugar starvation in Arabidopsis. Plant Cell Physiol. 48, 405–413 10.1093/pcp/pcm135 17234672

[B23] McClung C. R. (2006). Plant circadian rhythms. Plant Cell 18, 792–803 10.1105/tpc.106.040980 16595397 PMC1425852

[B24] McClung C. R. (2008). Comes a time. Curr. Opin. Plant Biol. 11, 514–520 10.1016/j.pbi.2008.06.010 18678522

[B25] Nikiforova V. J. Daub C. O. Hesse H. Willmitzer L. Hoefgen R. (2005). Integrative gene-metabolite network with implemented causality deciphers informational fluxes of sulphur stress response. J. Exp. Bot. 56, 1887–1896 10.1093/jxb/eri179 15911562

[B26] Rivals I. Personnaz L. Taing L. Potier M. C. (2007). Enrichment or depletion of a GO category within a class of genes: which test? Bioinformatics 23, 401–407 10.1093/bioinformatics/btl633 17182697

[B27] Rudelson M. Vershynin R. (2010). “Non-asymptotic theory of random matrices : extreme singular values,” in Proceedings of the International Congress of Mathematicians (Hindustan Book Agency), 1576–1602

[B28] Schaefer J. Strimmer K. (2005). A shrinkage approach to large-scale covariance matrix estimation and implications for functional genomics. Stat. Appl. Genet. Mol. Biol. 4, Article 32. 10.2202/1544-6115.1175 16646851

[B29] Smilde A. K. Westerhuis J. A. de Jong S. (2003). A framework for sequential multiblock component methods. J. Chemometr. 17, 323–337 10.1002/cem.811

[B30] Strayer C. Oyama T. Schultz T. F. Raman R. Somers D. E. Más P. (2000). Cloning of the arabidopsis clock gene TOC1, an autoregulatory response regulator homolog. Science 289, 768–771 10.1126/science.289.5480.768 10926537

[B31] Subramanian A. Tamayo P. Mootha V. K. Mukherjee S. Ebert B. L. Gillette M. A. (2005). Gene set enrichment analysis: a knowledge-based approach for interpreting genome-wide expression profiles. Proc. Natl. Acad. Sci. U.S.A. 102, 15545–15550 10.1073/pnas.0408351102 16199517 PMC1239896

[B32] ten Berge J. M. F. (1993). Least Squares Optimization in Multivariate Analysis. Leiden: DSWO Press

[B33] Thimm O. Bläsing O. Gibon Y. Nagel A. Meyer S. Krüger P. (2004). Mapman: a user-driven tool to display genomics data sets onto diagrams of metabolic pathways and other biological processes. Plant J. 37, 914–939 10.1111/j.1365-313X.2004.02016.x 14996223

[B34] Thioulouse J. Chessel D. Dolédec S. Olivier J.-M. (1997). ADE-4: a multivariate analysis and graphical display software. Stat. Comput. 7, 75–83 10.1023/A:1018513530268

[B35] Vershynin R. (2012). How close is the sample covariance matrix to the actual covariance matrix? J. Theor. Probab. 25, 655–686 10.1007/s10959-010-0338-z

[B36] Yeung K. Y. Ruzzo W. L. (2001). Principal component analysis for clustering gene expression data. Bioinformatics 17, 763–774 10.1093/bioinformatics/17.4.309 11590094

[B37] Zeeman S. C. Smith S. M. Smith A. M. (2007). The diurnal metabolism of leaf starch. Biochem. J. 401, 13–28 10.1042/BJ20061393 17150041

[B38] Zhou X. Jiang Y. Yu D. (2011). WRKY22 transcription factor mediates dark-induced leaf senescence in Arabidopsis. Mol. Cells 31, 303–313 10.1007/s10059-011-0047-1 21359674 PMC3933965

